# A comparative study of ^18^F-fluorodeoxyglucose positron emission tomography/computed tomography and ^99m^Tc-MDP whole-body bone scanning for imaging osteolytic bone metastases

**DOI:** 10.1186/s12880-015-0047-2

**Published:** 2015-03-01

**Authors:** Lin Zhang, Lihua Chen, Qiao Xie, Yongke Zhang, Lin Cheng, Haitao Li, Jian Wang

**Affiliations:** Department of Radiology, Southwest Hospital, Third Military Medical University, Chongqing, 400038 China; Department of Radiology, Taihu Hospital, Jiangsu Province, Wuxi, 214044 China

**Keywords:** Positron emission tomography, Radionuclide imaging, Skeleton, Metastatic tumor, Osteolytic

## Abstract

**Background:**

The objective of this study was to evaluate the feasibility and diagnostic value of ^18^F-fluorodeoxyglucose positron emission tomography/computed tomography (^18^F-FDG PET/CT) and ^99m^Tc-methylenediphosphonate (MDP) whole-body bone scanning (BS) for the detection of osteolytic bone metastases.

**Methods:**

Thirty-four patients with pathologically confirmed malignancies and suspected osteolytic bone metastases underwent ^18^F-FDG PET/CT and ^99m^Tc-MDP whole-body BS within 30 days. The sensitivity, specificity, and accuracy with respect to the diagnosis of osteolytic bone metastases and bone lesions were compared between the two imaging methods.

**Results:**

The sensitivity, specificity, and accuracy of ^18^F-FDG PET/CT for the diagnosis of osteolytic bone metastases were 94.3% (95% confidence interval [CI], 91.6–96.2%), 83.3% (95% CI, 43.6–96.9%), and 94.2% (95% CI, 91.5–96.1%), respectively. It was found that ^99m^Tc-MDP whole-body BS could discriminate between patients with 50.2% (95% CI, 45.4–55.1%) sensitivity, 50.0% (95% CI, 18.8–81.2%) specificity, and 50.2% (95% CI, 45.5–55.1%) accuracy. ^18^F-FDG PET/CT achieved higher sensitivity, specificity, and accuracy in detecting osteolytic bone metastases than 99mTc-MDP whole-body BS (p<0.001).

**Conclusions:**

F-FDG PET/CT has a higher diagnostic value than ^99m^Tc-MDP whole-body BS in the detection of osteolytic bone metastases, especially in the vertebra.

## Background

The skeletal system is one of the most common sites of malignant tumor metastasis. The early detection of bone metastases has significance in clinical staging, treatment, and prognosis [1,2]; imaging modalities that reveal these metastases, therefore, play an important clinical role. A number of different modalities have proven valuable in the detection of bone metastases; however, all non-invasive techniques currently in use have certain weaknesses [3]. ^99m^Tc- methylenediphosphonate (MDP) whole-body bone scanning (BS) is a conventional method used for the detection of bone metastases with a high sensitivity and at a low price. Early metastatic lesions may be missed; however, BS imaging relies on identifying an osteoblastic reaction rather than on the direct detection of tumor cells. Furthermore, low spatial resolution and low sensitivity to the treatment response restrict the use of BS [3]. When evaluating large numbers of suspected bone metastasis cases, bone scintigraphy is the most commonly used modality owing to its high sensitivity and availability, low cost, and the ease with which the entire skeleton can be surveyed. However, many patients with bone metastases do not show typical or specific patterns on scintigraphy scans [4]. It has been reported that ^18^F-fluorodeoxyglucose (FDG) positron emission tomography/computed tomography (PET/CT) has a different diagnostic value than ^99m^Tc-MDP BS with respect to malignant bone metastases. There is currently no consensus on the strengths and weaknesses of the two methods regarding the diagnosis of bone metastases [5-10]. The combined use of PET/CT and BS has been recommended for evaluating bone metastases in osteosarcoma patients [11]. Several studies have shown that PET achieves a higher sensitivity than BS when detecting bone metastases from sarcoma, whereas BS is superior (or at least not inferior) to PET in detecting bone metastases in the subgroup of patients with osteosarcoma [12]. Other studies have reported that the sensitivity of PET is lower than BS, and they suggest that PET can be used as a tool for confirming the positive results of conventional scans rather than as a means of initial detection [13,14]. Morris et al. [15] reported that bone scanning was significantly more sensitive (94%) than FDG (77%) in a series of 134 bone metastases. There is still no clear explanation for the differences between BS and ^18^F-FDG PET; consequently, we have undertaken a retrospective comparative study of ^18^F-FDG PET/CT and ^99m^Tc-MDP whole-body bone scan data. Data from 34 patients with pathology-proven malignancies and suspected osteolytic bone metastases were analyzed to determine the clinical value of the two imaging methods.

## Methods

### Patients

**Figure 1:**
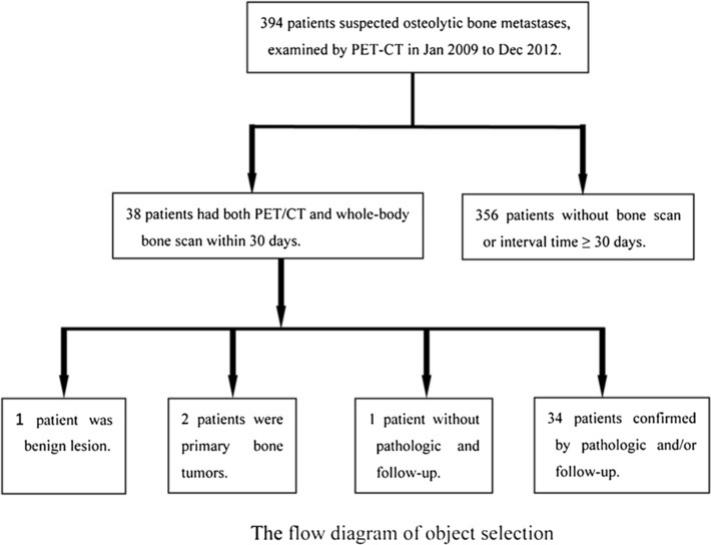
**Flow chart of the study profile according to the** ^**18**^ **F-FDG PET-CT and 99mTc-MDP whole-body bone scanning findings.**

The Medical Research Ethics Committee of the Third Military Medical University (Chongqing, China) reviewed and approved the present study. Informed consent was not required for this retrospective study. A total of 356 patients were examined using PET/CT between January 2009 and December 2012, and those meeting the following inclusion criteria were recruited to the study: had pathologically and follow-up confirmed malignancies and concurrent suspected osteolytic bone metastases; had no treatment before imaging; and had undergone PET/CT and ^99m^Tc-MDP BS procedures within 30 days of each other. A total of 34 patients (22 were male and 12 were female) were included in the study (Figure 1). In total, there were 21 cases of lung cancer, five cases of unknown primary tumor, two cases of lymphoma, and one case each of sarcoma, prostatic carcinoma, thyroid carcinoma, hepatoma, esophageal cancer, and gastric carcinoma.

### PET/CT scanning and 99mTc-MDP bone scans

PET-CT was performed using a Siemens Biography PET/CT scanner configured for two-slice spiral CT. The tracer agent used was ^18^F-FDG, and the radiochemical purity was ˃95%. All patients fasted for approximately 4–6 h before the scans, had blood glucose levels within the normal range and had urinated completely prior to scanning. An intravenous dose of ^18^F-FDG was administered at 0.1–0.15 mCi/kg. PET/CT imaging of the vertex to the upper thigh (five to six bed positions) was performed 60 min after ^18^F-FDG injection, and was then followed by an additional PET/CT scan of the lower extremities (six to seven bed positions). After locating the position and determining the range, all patients first underwent a whole body spiral CT transmission scan. The imaging parameters used for the CT scans were as follows: 140 kVp; 80 mA; 0.8 s/CT rotation; table speed, 22.5 mm/s; matrix, 512×512 (converted to 128×128 for image fusion); and a time of approximately 60 s. The CT data were used for attenuation correction, and the images were reconstructed using a conventional iterative algorithm. Immediately after CT scanning without intravenous iodinated contrast, PET data were acquired in the same anatomic locations using a two-dimensional model with a 128×128 matrix. The collection time was 3.5 min, with sections of 50 layers at every bed position and an iterative layer between two adjacent bed positions. We used ordered subset expectation maximization to reconstruct the image. The image was then transmitted to PACS, Fuison, and Esoft workstations to acquire transverse, sagittal, and coronary PET/CT images as well as the fused images.

BS was performed 3 h after intravenous injection of 925 MBq of ^99m^Tc-MDP using a Millennium MRP ECT (General Electric, Milwaukee, WI, USA). Anterior and posterior whole body planar images were acquired using a high-resolution collimator. Additional projections were obtained during individual examinations if indicated, but single-photon emission computerized tomography (SPECT) imaging was not performed.

### Image analysis

The results of PET/CT and BS were assessed by the consensus of two experienced nuclear medicine physicians who were aware of each patient’s history of malignancy but were blinded to the clinical findings, histopathological diagnosis, and other imaging data for each patient. All patient images were evaluated on a workstation computer using a DICOM viewer. In accordance with the literature, the skeletal system was divided into the following 13 skeletal areas: the cervical vertebra; thoracic vertebra; lumbar vertebra; sacrococcyx; left pelvis; right pelvis; left shoulder (including the scapula and clavicle); right shoulder; sternum; left rib; right rib; left limb; and right limb [16]. Visual observation and semi-quantitative analysis of ^18^F-FDG PET/CT images was performed using PET analysis software, which measured the maximum standardized uptake value (SUVmax) of ^18^F-FDG in the lesions. Based on the focal increased uptake of ^18^F-FDG PET/CT, the PET images were classified into one of the following three categories: positive; suspicious; and negative. The PET/CT images were classified as positive (metastasis) for bone metastasis based on the presence of newly detected, pathologically increased FDG uptake when compared with the normal bone surrounding the lesion, and a corresponding osteolytic change was observed in the CT images acquired during PET/CT. Any bone lesions that were detected during the CT component of PET/CT but that did not show FDG uptake were classified as negative. Increased ^18^F-FDG uptake was also considered negative if discovered in the joint or bone tissue surface. The rest of the cases were classified as suspicious [7,17]. BS images were used to classify tumors as negative (benign) or positive (metastasis) for bone metastasis, based on the new detection of pathologically increased MDP uptake as compared with the normal bone surrounding the lesions.

### Verification of osteolytic bone metastases

Osteolystic bone metastases were verified using one of the following methods: (a) histopathologically proven; and (b) based on a history of malignancy as well as X-ray and/or CT or magnetic resonance imaging (MRI) results, indicating obvious bone destruction without osteogenic imaging performance. The diagnosis could also be verified at follow-up if increased range and/or lesions of bone destruction were observed [9,17,18]. Clinical follow-up included PET/CT, BS, CT, MRI, and X-ray imaging for at least 6 months. Follow-up MRI, CT, and ^18^F-FDG PET procedures were performed in cases of very wide lesions with subsequent progression. Lesions exhibiting both osteolytic and osteosclerotic changes were considered verified by either type, depending on the predominant change in that lesion [4]. Any of the following findings were considered false positives: (a) benign lesions identified in the postsurgical specimen; (b) positive lesions initially revealed using imaging but that decreased in size or showed no significant changes for at least 6 months; and (c) lesions that resolved spontaneously [11].

### Statistical analysis

The number of abnormal bone changes detected using PET/CT and BS in the same field of view was compared. Additionally, the diagnoses made using both modalities were compared with the final diagnoses. The sensitivity, specificity, and accuracy of ^18^F-FDG PET/CT and ^99m^Tc-MDP BS were compared using McNemar’s test; results were considered statistically significant when the P value was ˂0.001. The Kappa test was used to analyze and compare the consistency of lesion detection using the two modalities; the differences were statistically significant when Kappa was <0.05.

## Results

**Figure 2:**
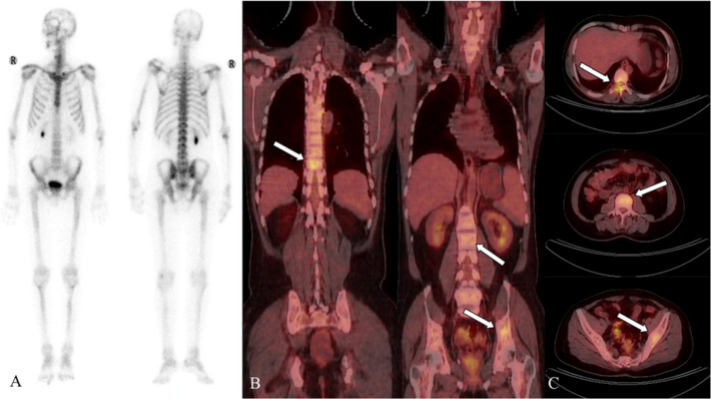
**A 32-year-old man with an unknown primary lesion and multiple bone metastases.** There was no focally increased uptake detected in the bone scanning (BS) image **(A)**. PET-CT revealed that the bone substance of the ninth thoracic vertebral body and its adjunct, the third lumbar vertebral body and left ilium, was destroyed. ^18^FDG uptake in these areas was significantly increased. The maximum standardized uptake value is 3.96 **(B and C)**. More metastatic lesions were detected using ^18^F-FDG PET-CT than 99mTc-MDP BS.

The characteristics of all of the included patients are detailed in Table 1. The median age of the patients was 57 (range, 25–77) years. A total of 422 skeletal areas were analyzed for lesions in 34 patients. In total, 405 lesions were confirmed as osteolytic metastases. The distribution and number of lesions are shown in Table 2. The two imaging methods that we evaluated were better at detecting lesions, and a greater number of malignant lesions were revealed using ^18^FDG-PET/CT (Figure 2; Table 1). There was a significant difference in the number of lesions detected in different skeletal areas using PET/CT and BS. In total, ^18^F-FDG PET/CT was used to detect 383 positive lesions and 23 false-negative lesions, yielding a sensitivity of 94.3% (95% confidence interval [CI], 91.6–96.2%); BS was used to detect 204 positive lesions and 202 false-negative lesions, yielding a sensitivity of 50.2% (95% CI, 45.4–55.1%). Using PET/CT imaging, five negative lesions and one false-positive lesion was identified among the six benign lesions, while the BS images indicated three negative and three false-positive lesions; the specificities of the two methods were 83.3% (95% CI, 43.6–96.9%) and 50.0% (95% CI, 18.8–81.2%), respectively. The diagnostic accuracies of ^18^F-FDG PET/CT and ^99m^Tc-MDP whole-body BS were 94.2% (95% CI, 91.5–96.1%) and 50.2% (95% CI, 45.5–55.1%), respectively. There were significant differences in the sensitivity, specificity, and accuracy (all p<0.001) of these modalities, indicating that ^18^F-FDG PET/CT was more accurate than BS in the present study (Table 2). ^18^F-FDG PET/CT showed higher sensitivity than 99mTc-MDP BS in detecting osteolytic bone metastases in the cervical vertebra, thoracic vertebra, lumbar vertebra, and ribs (Table 3; all p<0.001). The subgroups were created in terms of tumor types and bone areas; the sensitivity estimates for the different subgroups are presented in Table 4.

**Table 1:** **Patient characteristics**

**Category**	**Number of people**	**Proportion**
Gender		
Male	22	64.7%
Female	12	35.3%
Tumor types		
Lung cancer	21	61.9%
Lymphoma	2	5.9%
Sarcoma	1	2.9%
Prostatic carcinoma	1	2.9%
Thyroid carcinoma	1	2.9%
Hepatoma	1	2.9%
Esophagus cancer	1	2.9%
Gastric carcinoma	1	2.9%
Unknown primary tumor	5	14.8%

**Table 2:** **Comparison of the performance of** ^**18**^ **F-FDG PET-CT and** ^**99m**^ **Tc-MDP whole-body bone scanning in the detection of osteolytic bone metastases**

	**Sensitivity (%)**	**Specificity (%)**	**Accuracy (%)**
^18^F-FDG PET-CT	94.3 (91.6, 96.2)	83.3 (43.6, 96.9)	94.2 (91.5, 96.1)
^99m^Tc-MDP	50.2 (45.5, 55.1)	50.0 (18.8, 81.2)	50.2 (45.5, 55.1)
p value	<0.001	<0.001	<0.001

**Table 3:** **The number of osteolytic bone metastases in the 13 bone areas and the number of detected lesions using** ^**18**^ **F-FDG PET-CT and** ^**99m**^ **Tc-MDP whole-body bone scanning**

**Bone area**	**Number of lesions**	**Detected lesions**		**P value**
		**PET-CT**	**Bone scan**	
Cervical vertebra	32	32	7	<0.001
Thoracic vertebra	102	99	48	<0.001
Lumbar vertebra	52	49	21	<0.001
Sacrococcyx	10	10	4	0.094
Sternum	5	5	4	0.221
Left pelvis	23	21	14	0.099
Right pelvis	20	17	10	0.043
Left shoulder	7	6	4	0.554
Right shoulder	8	6	6	0.999
Left rib	55	52	35	<0.001
Right rib	46	46	23	<0.001
Left limb	18	16	12	0.229
Right limb	28	24	16	0.038
Total	406	383	204	<0.001

**Table 4:** **Comparison of the sensitivity of** ^**18**^ **F-FDG PET-CT and 99mTc-MDP whole-body bone scanning for different tumor types and bone regions**

**Subgroup**	^**18**^ **F-FDG PET-CT**	^**99m**^ **Tc-MDP**	**P value**
Tumor types			
Lung cancer	92.9 (88.6, 95.6)	58.3 (51.6, 64.7)	<0.001
Other types	96.3 (92.7, 98.2)	54.6 (47.6, 55.9)	<0.001
P value	0.182	0.835	-
Bone areas			
Vertebra	96.6 (93.9, 98.1)	45.5 (39.9, 51.1)	<0.001
Other areas	88.9 (81.6, 93.5)	64.8 (55.4, 73.1)	<0.001
P value	0.167	<0.001	-

Note: −, no value.

**Figure 3:**
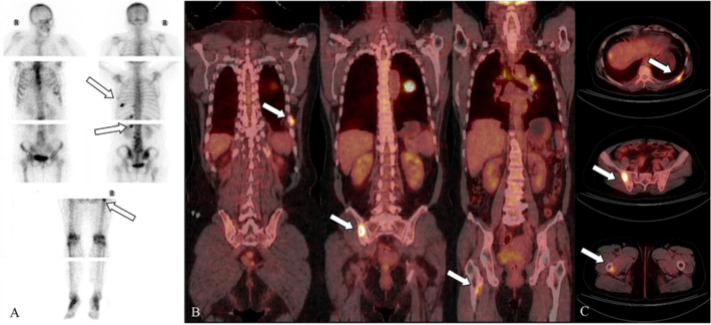
**A 54-year-old woman with left lung cancer.** Bone scanning (BS) shows focally increased uptake in the left ninth, tenth, and twelfth posterior ribs, three to five lumbar vertebrae and nearby regions of the lesser trochanter of the right femur **(A)**. PET-CT revealed increased 18FDG intake at the left tenth posterior rib, right ilium, and right femur; the maximum standardized uptake value was 12.6 **(B and C)**. More metastatic lesions were detected using 99mTc-MDP BS than 18F-FDG PET-CT.

**Figure 4:**
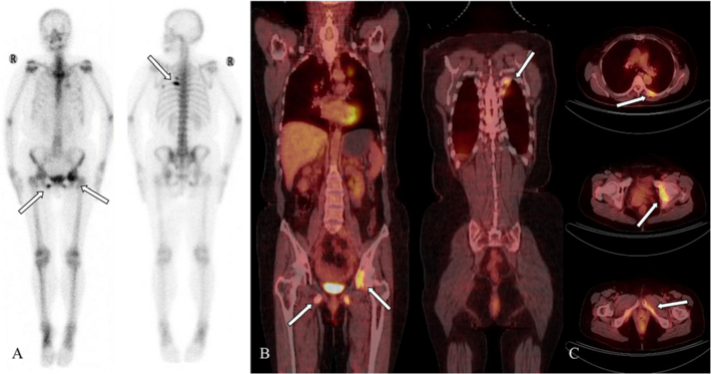
**A 61-year-old woman with left superior lung cancer.** Bone scanning (BS) indicated focally increased uptake in the left fifth and sixth posterior rib, the left acetabulum, and the bilateral inferior ramus of the pubis **(A)**. PET-CT revealed that the bone in the left fifth and sixth posterior ribs, the left acetabulum, and the bilateral inferior ramus of the pubis was destroyed. ^18^FDG uptake in these areas was significantly increased. The maximum standardized uptake value was 5.25 **(B and C)**. The same number of metastatic lesions were detected using PET-CT and BS.

The diagnoses made using ^18^F-FDG PET/CT and BS are described in Table 5. The number of positive and negative lesions identified using ^18^F-FDG PET/CT were 388 and 319, respectively; ^99m^Tc-MDP BS images revealed 207 positive and 501 negative lesions. There was no significant difference in the diagnostic consistency of the two methods (p<0.001); their consistency was poor as shown in Figures 3 and 4.

**Table 5:** **Comparison of diagnostic consistency using** ^**18**^ **F-FDG PET-CT and 99mTc-MDP whole-body bone scanning**

**Bone scan**	**PET-CT**	
	**+**	**-**
+	171	36
-	217	283

Note: p<0.001.

## Discussion

Malignant tumors usually metastasize to the skeletal system. The site of metastasis and quantity of metastatic lesions are closely related to the therapeutic regimen and prognosis. Clinically, ^99m^Tc-MDP is more frequently used for the diagnosis of bone metastases, and it is successful. However, ^99m^Tc-MDP is less sensitive to purely lytic lesions. MDP uptake is not limited to malignant tumors and can also take place in benign lesions [19]. Planar bone scan images provide amorphous information and cannot be used to reliably determine the exact location of bone lesions.

The ^18^F-FDG PET/CT modality, which uses ^18^F-FDG as a tracer agent to display lesions, has been widely used worldwide. The CT components of PET/CT procedures can be used to precisely locate metastatic sites and identify morphological changes as being osteoblastic or lytic. CT is also a vital choice in the initial evaluation of the risk of bone fractures [20]. BS and SPECT identify osteoblastic responses, while ^18^F-FDG uptake detected using PET is related to increased intratumoral glycolysis [21]. Shie et al. [20] performed a meta-analysis comparing the diagnostic accuracy of ^18^F-FDG-PET and BS in breast cancer bone metastasis. The authors reported an overall PET sensitivity of 81% (95% CI, 70–89%), a specificity of 93% (95% CI, 84–97%), and an area under the curve (AUC) of 0.08; the overall sensitivity of BS was 78% (95% CI, 67–86%), the specificity was 79% (95% CI, 40–95%), and the AUC was 0.43%. The results of their analysis suggested that it remains unclear as to whether PET or BS is superior regarding the diagnosis of breast cancer bone metastases [20]. However, the specificity of PET is higher; as a method for a certain diagnosis, PET could be more useful for monitoring therapeutic effects.

By analyzing and comparing the use of ^99m^Tc-MDP BS and ^18^F-FDG PET/CT to diagnose osteolytic bone metastasis, modalities that focus specifically on lesions or skeletal areas, we may safely draw conclusions regarding their effectiveness. With respect to osteolytic lesions, the sensitivity, specificity, and accuracy of ^18^F-FDG PET/CT were 94.3% (95% CI, 91.6–96.2%), 83.3% (95% CI, 43.6–96.9%), and 94.2% (95% CI, 91.5–96.1%), respectively; the sensitivity, specificity, and accuracy of ^99m^Tc-MDP BS were 50.2% (95% CI, 45.4–55.1%), 50.0% (95% CI, 18.8–81.2%), and 50.2% (95% CI, 45.5–55.1%). The differences in the sensitivity, specificity, and accuracy between the two modalities were all significant. The sensitivity and specificity of ^18^F-FDG PET/CT were higher than ^99m^Tc-MDP BS for osteolytic bone metastases in the cervical vertebra, thoracic vertebra, lumbar vertebra, and the left and right rib. These results are consistent with those of other studies. ^18^F-FDG PET is expected to have better sensitivity and specificity than SPECT or BS because the modality directly images tumor metabolism using ^18^F-FDG; tumors can therefore be identified before sufficient ^99m^Tc-MDP has accumulated for it to be detected using BS [22]. As compared with ^99m^Tc-MDP bone scanning, ^18^F-fluoride PET/CT was found to be superior in terms of all measured parameters when used to detect prostate and breast cancer, but the sensitivity and negative predictive value (NPV) of these modalities were equal in non-small-cell lung cancer. Additionally, MDP BS had a superior sensitivity and NPV relative to FDG PET/CT, but had low specificity and positive predictive value [19].

The consistency of the diagnostic results regarding the two methods was poor, and possible reasons are as follows. (a) PET/CT and BS rely on the uptake activity of different radiotracer molecules regarding the identification of osteolytic bone metastases. ^18^F-FDG PET/CT reveals the metabolic activity of cells based on glycometabolic changes in tissue. In cases of osteolytic bone metastases, ^18^F-FDG uptake is higher in tumors than in the surrounding normal tissue. In contrast, ^99m^Tc-MDP uptake is usually high in bone, but the metastases cause ossification and increased local blood flow. This results in decreased uptake of the bone-imaging agent; BS is then used to detect lesions by identifying areas with significantly lower uptake density [9]. (b) Bone metastases commonly originate from the medulla and then destroy the cortical bone outwards. When a lesion is located in the medulla, the metabolism of local bone tissue increases and, consequently, so does ^18^F-FDG uptake. However, at this point in a tumor’s development it is difficult to detect using ^99m^Tc-MDP BS. (c) Particularly with respect to CT images, the spatial resolution of PET/CT is significantly higher than BS images. In cases of smaller osteolytic lesions, the detection ratio is obviously higher using PET/CT than using BS [8]. (d) PET/CT can be used to examine the whole body systematically and comprehensively, making it possible to identify primary tumors and/or lesions throughout the body, which is useful for distinguishing between benign and malignant lesions. This feature affects the specificity and accuracy of the modality in cases of osteolytic bone metastases.

The present study had several limitations apart from its retrospective design. First, not all suspected lesions were histologically confirmed. Second, there was a limited number of cases and the primary tumor types included no osteoblastic or mixed bone metastases. BS is very sensitive to osteoblastic activity and can be used to detect a 5–10% change in the blastic response [23]. However, ^18^F-FDG SUVs were lower in sclerotic lesions when compared with lytic lesions [24]. According to a study by Nakai et al. [25] involving the evaluation of breast cancer bone metastasis, the following three types of lesions can be distinguished using CT: osteoblastic; osteolytic; and no-change. These authors reported osteoblastic, osteolytic, and no-change lesion detection rates of 100%, 70.0%, and 25.0%, respectively, using BS, and 55.6%, 100%, and 87.5%, respectively, using PET/CT [25]. It is far from certain which method has superior diagnostic performance in cases of bone metastases. Thus, the deviation in the statistical results is inescapable. The exact differences between BS and PET/CT detection of osteoblastic or mixed bone metastases require further investigation. Third, we did not analyze the risk of radiation exposure. Fourth, no additional SPECT or SPECT/CT imaging was performed in our study. However, the use of SPECT/CT with planar imaging can improve diagnostic confidence or identify additional metastatic lesions [26]. As a planar acquisition, SPECT has been reported to enhance the quality of planar scintigraphy, in particular improving spatial resolution. The addition of SPECT makes the test marginally more sensitive, but specificity reportedly increases by 25–30 [27]; in addition, SPECT allows some measure of anatomic localization. However, the drawback of SPECT is that it can only be used in a limited area [19]. Because of our limited data, we did not assess the performance of SPECT in the current study. Additionally, our ^18^F-FDG PET/CT images spanned the skull to the mid-thigh, and not the whole body, which might have caused false negatives in the lower extremities. Finally, we did not analyze the cost effectiveness of these modalities. The exact differences between BS and PET/CT in diagnosing bone metastasis need further investigation.

## Conclusions

When detecting osteolytic bone metastases, ^18^F-FDG PET/CT has a higher diagnostic value than ^99m^Tc-MDP BS; in clinical practice, physicians should choose the most accurate method. BS remains the primary method for the initial diagnosis and screening of osseous metastasis, although its diagnostic role and future application may be partially replaced by powerful new imaging methods.

## Abbreviations

FDG: Fluorodeoxyglucose
PET/CT: Positron emission tomography/computed tomography
SUVmax: Maximum standardized uptake value
BS: Bone scanning
SPECT: Single photon emission computerized tomography
SUV: Standardized uptake value
MDP: Methylenediphosphonate
MRI: Magnetic resonance imaging
AUC: Area under the curve
NPV: Negative predictive value
